# Mifepristone Treatment during Early Adolescence Fails to Restore Maternal Deprivation-Induced Deficits in Behavioral Inhibition of Adult Male Rats

**DOI:** 10.3389/fnbeh.2016.00122

**Published:** 2016-06-15

**Authors:** Jiska Kentrop, Liza van der Tas, Manila Loi, Marinus H. van IJzendoorn, Marian J. Bakermans-Kranenburg, Marian Joëls, Rixt van der Veen

**Affiliations:** ^1^Department of Translational Neuroscience, Brain Center Rudolf Magnus, University Medical Center UtrechtUtrecht, Netherlands; ^2^Centre for Child and Family Studies, Leiden UniversityLeiden, Netherlands

**Keywords:** early life stress, maternal deprivation, adolescence, mifepristone, impulsivity, attention, 5-choice serial reaction time task

## Abstract

Early life adversity has a profound impact on brain development and later life health. Animal models have provided insight how early life stress programs stress responsiveness and might contribute to the development of psychiatric disorders. In the present study, the long-term effects of maternal deprivation (MD) on behavioral inhibition and attention were examined in adult male Wistar rats. To this end animals were tested in the 5-choice serial reaction time task (5-choice SRTT). We also explored the potential of a 3-day treatment with the glucocorticoid receptor (GR) antagonist mifepristone during early adolescence to normalize putative behavioral effects of early life stress. Deprivation of the mother for 24 h on postnatal day (PND) 3 led to a modest but significant increase in premature responses in the 5-choice SRTT, but did not affect measures of attention. Body weight was lower in deprived animals from weaning until the start of testing. Early adolescent mifepristone treatment (PND 26–28) did not influence performance on the 5-choice SRTT and did not mitigate the deprivation-related impairment in behavioral inhibition. Our results indicate that MD leads to impaired behavioral inhibition, and that mifepristone treatment during early adolescence does not normalize the behavioral changes caused by early life stress.

## Introduction

Early life adversity has a profound impact on later life health. Growing up in stressful early life conditions has been associated with a higher incidence of psychiatric disorders, including mood and anxiety disorders (Carr et al., 2013; Nelson et al., 2014), as well as impulsivity and attention related disorders, such as substance abuse, addictive disorders, schizophrenia and attention deficit/hyper activity disorder (ADHD; Laurent et al., 1999; Tarter et al., 2004; Biederman, 2005; Lovallo, 2013; Babenko et al., 2015). Animal models of early life stress have provided valuable insights into the role of hypothalamus-pituitary-adrenal (HPA) axis programming in the development of these disorders.

Important aspects of the early life environment of rodents are mother-pup interactions and interactions with littermates. In the first 2 weeks of life, the rodent HPA-axis is characterized by stress hyporesponsivity. However, when pups are deprived from the dam in this period for a single prolonged period of time (maternal deprivation [MD]) or repeatedly separated from the dam for at least 1 h per day (maternal separation), this causes a rise in corticosterone level that is thought to affect HPA-axis programming for life (Lehmann and Feldon, 2000; Levine, 2005). Corticosterone exerts its effects via the mineralocorticoid receptor (MR) and glucocorticoid receptor (GR), which act in balance to regulate the stress response (de Kloet et al., 2005). It is thought that early life adversity can cause a MR:GR imbalance, leading to HPA-axis dysregulation with long-lasting consequences for stress responsiveness and emotional and cognitive functioning (de Kloet et al., 1998, 2005; Oitzl et al., 2010; Krugers and Joëls, 2014).

Indeed, several studies showed that rats subjected to MD or maternal separation have increased basal corticosterone levels at the age of 3 months (Lehmann and Feldon, 2000; Workel et al., 2001; Lehmann et al., 2002) and exhibit HPA-axis hyperactivity in response to acute stress in adulthood (Plotsky and Meaney, 1993; Aisa et al., 2007). Rodents with a history of early life adversity display more anxiety-related behavior in the elevated plus maze, reduced feeding in a novel environment and reduced activity in an open field (Wigger and Neumann, 1999; Macrí et al., 2004; Aisa et al., 2007; Pascual and Zamora-León, 2007; Li et al., 2013). Increased depressive-like behavior in the forced swimming test and sucrose intake test are also commonly reported (Morley-Fletcher et al., 2003; Cui et al., 2006; Aisa et al., 2007; Van Waes et al., 2011). In the cognitive domain, maternal separation during the first 3 weeks of life impairs learning of male Wistar rats in the Morris water maze and novel object recognition test (Aisa et al., 2007). Similarly, impaired learning in the Morris water maze was observed in rats earlier subjected to 24 h MD on postnatal day 3 (PND 3; Oomen et al., 2010).

Far less is known about the effects of early life adversity on impulsivity and the effects reported thus far are inconsistent. Impulsivity, or the lack of behavioral inhibition, can be defined as the tendency to act prematurely without foresight and can be subdivided in impulsive choice and impulsive action (Dalley et al., 2008). Impulsive choice involves the preference for immediate small rewards over delayed larger rewards, while impulsive action reflects the failure to inhibit a response once it is initiated. Adolescent rats subjected to 24 h MD on PND 9 showed increased impulsive choice in the intolerance-to-delay task (Marco et al., 2007). Fuentes et al. (2014), however, using a combination of maternal separation (1 h/day PND 1–8) and restriction of bedding material, found decreased impulsive choice in females, and no effect in males in a delay discounting paradigm. They found no effect on behavioral inhibition during acquisition of the 5-choice serial reaction time task (5-choice SRTT; Fuentes et al., 2014). Rats exposed to a paradigm of severe separation stress consisting of separation from the mother and siblings from PND 2 onwards, showed increased impulsive action, but no change in impulsive choice in adulthood (Lovic et al., 2011a). Housing rats in a complex rearing environment during adolescence in combination with a history of early life MD impaired behavioral inhibition but improved attention (van der Veen et al., 2015). MD by itself did not significantly affect behavioral inhibition, but this could be explained by the fact that the effects of complex housing were so prominent that potential effects of MD were possibly masked.

Therefore we now set out to specifically study the long-term effects of 24 h MD at PND 3 on behavior in the 5-choice SRTT, an operant task in which both attention and behavioral inhibition can be measured (Robbins, 2002). Given the effect of early life stress on HPA-axis dysregulation, we were also interested in the ability of the non-selective GR antagonist mifepristone to reverse potential effects of early life stress on behavior. Acute administration of mifepristone in adult rats has previously been found to reverse the reduction in adult neurogenesis caused by chronic stress or chronic corticosterone treatment (Mayer et al., 2006; Oomen et al., 2007; Hu et al., 2012). Moreover, acute mifepristone treatment in adulthood was also found to completely reverse maternal separation induced impairments in the forced swimming test and novel object recognition test (Aisa et al., 2007, 2008). We reasoned that the effects of acute mifepristone administration on behavior might be particularly effective when brain areas involved in impulsive behavior are still highly plastic, such as during (early) adolescence. We therefore aimed to reverse putative behavioral effects of MD through a 3-day early adolescent treatment with mifepristone between PND 26 and 28. This period, the start of adolescence, has been characterized as a sensitive period for HPA-axis manipulations (Romeo, 2010). Treatment with mifepristone between PND 26 and 28 was previously shown to be effective in reversing the reduction in neurogenesis after MD (Loi et al., 2014) as well as the behavioral effects of MD on reward-based decision making (M. Loi, personal communication). We hypothesized that attention and behavioral inhibition, critically depending on PFC functioning, are affected by MD and sensitive to mifepristone treatment during early adolescence.

## Materials and Methods

### Animals

Male and female Wistar rats were obtained at 6 weeks of age (Charles River Laboratories, Arbresle, France). Animals were kept in a temperature (21°C) and humidity (55%) controlled room with a 12 h light/dark cycle (lights on at 7:00 am). Breeding started after animals had been familiarized with our animal facility for at least 3 weeks. Food and water was available *ad libitum*. For this experiment, we used the offspring of 16 dams, equally distributed over the experimental groups. Only male offspring (*n* = 48) was used for testing. Testing started at 90 days of age when animals weighed on average 330 g. Three weeks before testing, the light/dark cycle was reversed (lights off at 7:00 a.m.) to assure that animals were tested in their active phase. One week before testing males were gradually food deprived (16 g of chow a day) until they reached 90–95% of their free-fed weight and they were kept within this range throughout testing. Once a week cages were cleaned and general health status was checked. Some aspects of the experimental protocol have been described elsewhere in more detail (van der Veen et al., 2015). Experiments were approved by the local committee for Animal Health, Ethics and Research of Utrecht University. Animal care was conducted in accordance with the EC Council Directive of November 1986 (86/609/EEC).

### Early Life Experience: Breeding and Maternal Deprivation

Two females were paired with a male for 10 days. The females stayed together for another week and were then individually housed to prepare for birth. A paper towel was provided to the mothers as nesting material. At PND 3, dams were taken out of their home cage and placed in another cage. The sex of the pups was determined and when necessary litters were sex-balanced and culled to a minimum of 6 and a maximum of 10 pups. Litters were randomly assigned to the MD or control condition. Mothers in the control group were placed back into their home cage within 2 min, while for the experimental group MD started. During MD, litters stayed together in their home cage (without the dam) and were transported to an adjacent room. The cage was placed on a heating plate (33°C) to prevent hypothermia of the pups. After 24 h the cage was taken back to the original room and the mother was reunited with her litter.

### Early Adolescence: Weaning and Mifepristone Treatment

Pups were weaned at 21 days of age and were housed in pairs in standard Makrolon cages (37 × 20 × 18 cm). Rats were randomly assigned to the mifepristone or vehicle treatment groups. At PND 26, 27 and 28 rats were twice daily treated with either the GR antagonist mifepristone (RU38486, kindly provided by Corcept Pharmaceuticals, CA, USA) or vehicle. Mifepristone treatment consisted of 5 mg mifepristone/100 g body weight, dissolved in 15 μL 99% ethanol and 1.25 mL coffee cream (Campina, Woerden, Netherlands). Vehicle treatment consisted of coffee cream and ethanol administered in the same volumes as described for mifepristone treatment. Pups from the same mother were placed in both the experimental (mifepristone) and control group (vehicle) to minimize litter effect. No more than two pups from the same mother were placed in the same experimental condition.

### Testing: 5-choice Serial Reaction Time Task (5-choice SRTT)

Four different groups were tested in the 5-choice SRTT: non-maternally deprived males, either treated with vehicle (No-MD Veh, *n* = 8) or mifepristone (No-MD Mif, *n* = 16), and maternally deprived males treated with vehicle (MD Veh, *n* = 8) or mifepristone (MD Mif, *n* = 16). Daily sessions were performed during the dark phase (Monday to Friday), using procedures adapted from Robbins (2002) and Bari et al. (2008). Throughout the 5-choice SRTT experiment animals were pair-housed.

#### Apparatus

The 5-choice SRTT was conducted in operant conditioning chambers (Med Associates, St. Albans, VT, USA). Each chamber (30.5 × 24.1 × 21 cm) was located within a larger exterior opaque box equipped with exhaust fans that assured air renewal and masked background noise. The rear wall of the chamber was curved and contained a set of five holes, each equipped with an infrared detector and a yellow light emitting diode stimulus light. Sucrose pellets (45 mg, Formula P; Bio-Serv) were delivered at the opposite wall, in a larger pellet magazine, also equipped with infrared detectors. A white house light, located at the roof, could be switched on. Experimental contingencies were controlled and data were collected using MED-PC version 14.0 (Med Associates).

#### Habituation and Pellet Magazine Training

During the first 2 days, animals were habituated to the chambers for 20 min. Sucrose pellets were placed in all five response holes and in the pellet magazine. Habituation was followed by two magazine training sessions, where 80 sucrose pellets were delivered in the pellet magazine within 20 min, with an average interval of 15 s.

#### Training 5-choice Task

Rats were trained to respond to a brief visual stimulus presented randomly in one of the five nose poke apertures to obtain a sucrose pellet. Each training session started with a 2 min habituation period in which no reward could be obtained (house light switched on). Then the house light was switched off, a “free” pellet was given, and the rat initiated the first trial by collecting this pellet in the pellet hole. On the start of a trial, one stimulus hole was illuminated. With a nose entry into this hole, a sucrose pellet was released into the pellet hole. After collecting this pellet, an inter-trial interval (ITI) of 5 s (ITI5) started, followed by the next trial. A session ended when 100 trials had been accomplished or 30 min had elapsed. In phase I of training, all five stimulus lights were “ON” at the start of a trial and a nose entry in either hole released a sucrose pellet. Animals were trained until each rat obtained 100 pellets (all within 4 days). Starting *phase II* *of training*, stimulus holes were illuminated in a pseudorandom order and each hole was illuminated 20 times during a 100 trials session. In phase II of training a stimulus hole was illuminated until nose entry. Entries in other (unlit) holes were counted, but without consequences. All animals obtained 100 pellets within 3 days. In phase III of training stimulus time was gradually decreased (16, 8, 4, 2, 1.5, 1.2 s) to reach the training endpoint of 1.2 s. The rats had a limited time to respond to the stimulus (limited hold = stimulus time +2 s, with a minimum of 5 s. In this stage of training, an omission (no response), premature response (response in the ITI) or incorrect response (response in unlit hole) resulted in a time-out period. During this time-out period, no reward could be obtained (house light switched on). Responding in stimulus holes during time-out resulted in a reset of the time-out period. Animals were trained on each stimulus duration until they finished 100 trials in 30 min with a performance accuracy >80% (correct choice) and errors of omission <20. Training was completed when animals reached stable baseline responding at 1.2 s stimulus duration over at least three consecutive training days. Apart from habituation and time-out periods, the house light was switched off during the test in order to increase the contrast for visual discrimination of the stimulus lights for the Albino Wistar rat.

#### Behavioral Inhibition and Attention in the 5-choice Task

Behavioral inhibition, i.e., the ability to withhold responding, was challenged in two ways: (1) Lengthening the ITI to 7 s; and (2) using a random ITI (5, 7, 10, 13 and 15 s). The level of sustained (spatial) attention was investigated by increasing attentional load via: (1) shortening the stimulus time to 0.5 s; or (2) introducing a novel object in the cage. This object was a wooden block of 3 cm high covering the middle line of the chamber and providing a light hurdle between stimulus holes and pellet hole. Between test sessions, baseline sessions (ITI5, 1.2 s stimulus duration) were performed until stable responding was resumed. The following measures were recorded: (1) *Accuracy*: percentage of correct responses [(correct/correct + incorrect) × 100]; (2) *Omissions*: number of missed trials; (3) *Latency to correct*: latency between stimulus presentation and correct choice; (4) *Latency to reward*: latency to collect the reward after correct choice; (5) *Premature responses*: number of nose pokes before the presentation of the stimulus light; (6) *Perseverative responses*: number of nose pokes after correct choice, a measure related to compulsive behavior. In addition, behavior in no-reward periods was recorded; (7) Number of nose pokes in the pellet hole during ITIs (NP pellet hole ITI); and (8) frequency of nose pokes in pellet hole and stimulus holes, respectively, per time-out period (NP pellet hole/TO and NP stimulus holes/TO). Since the number of time-out periods varied between animals, behavior during this “punishment” period was computed as behavior per time-out.

#### Statistical Analyses

Statistical analyses were performed using SPSS for windows version 23 (IBM, NY, USA).

Data are presented as Mean ± SEM. Early life experience (no-MD vs. MD) and early adolescent treatment (Veh vs. Mif) served as between-subject factors. An independent *t*-test was used to assess the effect of MD on PND 26 body weights (before the start of mifepristone treatment). Univariate analysis of variance (ANOVAs) were performed to compare group differences in body weight at 12 weeks of age. To compare experimental groups on habituation, repeated measures ANOVAs were performed over 5 min blocks. Univariate ANOVAs were performed to compare group differences at the end of training. Responses in the testing conditions (ITI 7 s, ITI random, stim 0.5 s and novel object) were compared to responses during baseline (end of training) in repeated measures ANOVAs with ITI 7 s, random ITI, 0.5 s stimulus duration and novel object as within-subject factors where applicable.

For every statistical test that is reported, the first result is presented with *F* or *t* value to include the degrees of freedom. Any further results of the same statistical test were reported with *p* and $\eta_{p}^{2}$ only. Not all animals reached 100 trials in the ITI7 and random ITI test conditions. Responses were therefore also calculated per trial. Performance per trial was highly correlated with total performance (*r*s ranging between 0.89–1.00, *p* < 0.001), and results of the analyses were similar. We here only report on total performance.

Outlying scores (>3.29 standard deviations (SD) above the mean), were substituted with the next highest score (winsorized, Tabachnick and Fidell, 2006) to mitigate excessive influence of outliers without excluding subjects. In total 11 data points were winsorized (in eight different measures). Of note, if these outliers were excluded, this did not change any of the outcomes.

## Results

### Body Weight

**Figure 1:**
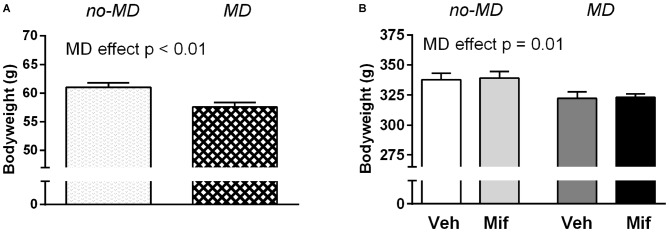
**The effect of maternal deprivation (MD) and mifepristone treatment (Mif) on body weight. (A)** Body weight at postnatal day 26 (PND 26), before the start of mifepristone/vehicle (Veh) treatment. Group sizes: No-MD: *n* = 24; MD: *n* = 24. **(B)** Body weight at 12 weeks, just before the start of the 5-choice experiment. Group sizes: No-MD Veh: *n* = 8; No-MD Mif: *n* = 16; MD Veh: *n* = 8; and MD Mif: *n* = 16. Graphs represent Mean ± SEM. At PND 26, MD rats weighed less than no-MD rats (*t*_(46)_ = 3.22, *p* < 0.01, $\eta_{p}^{2}$ = 0.18; **A**) and this difference persisted until the start of 5-choice SRTT training at week 12 (*F*_(1,44)_ = 8.93, *p* = 0.01, $\eta_{p}^{2}$ = 0.17; **B**).

Body weight was first measured on PND 26, just before the start of Mif treatment (Figure 1A). MD rats weighed less than no-MD rats (*t*_(46)_ = 3.22, *p* < 0.01, $\eta_{p}^{2}$ = 0.18). This difference in body weight was still visible at the start of the 5-choice SRTT training, i.e., before food restriction started, at week 12 (*F*_(1,44)_ = 8.93, *p* = 0.01, $\eta_{p}^{2}$ = 0.17; Figure 1B). Mif treatment by itself did not affect body weight (*p* = 0.83, $\eta_{p}^{2}$ = 0.00), nor did it moderate the effect of MD on body weight (*p* = 0.96, $\eta_{p}^{2}$ = 0.00).

### Habituation to the 5-choice Chamber and Acquisition of the Task

All animals habituated to the test chamber, as evident from a strong decrease in nose pokes over time during the first habituation session of 20 min (*F*_(3,132)_ = 28.92, *p* < 0.001, $\eta_{p}^{2}$ = 0.40; data not shown). MD did not influence habituation (*p* = 0.21, $\eta_{p}^{2}$ = 0.03). Mif treatment did also not affect habituation (*p* = 0.67, $\eta_{p}^{2}$ = 0.01) and there was no interaction between the two treatments (*p* = 0.21, $\eta_{p}^{2}$ = 0.04).

Speed of learning was assessed by comparing accuracy and number of omissions between groups on the first days of training when the stimulus light decreased in duration (16–1.5 s). Overall, accuracy increased over the course of training, (*F*_(4,176)_ = 6.38, *p* < 0.001, $\eta_{p}^{2}$ = 0.13). The number of omissions increased when stimulus duration was shortened (*F*_(4,176)_ = 114.36, *p* < 0.001, $\eta_{p}^{2}$ = 0.72). The speed of learning was not influenced by MD (accuracy: *p* = 0.48, $\eta_{p}^{2}$ = 0.02; omissions: *p* = 0.62, $\eta_{p}^{2}$ = 0.01; data not shown). Mif treatment did also not influence the speed of learning (accuracy: *p* = 0.34, $\eta_{p}^{2}$ = 0.03; omissions: *p* = 0.51, $\eta_{p}^{2}$ = 0.02), nor was there an interaction between MD and Mif treatments (accuracy: *p* = 0.38, $\eta_{p}^{2}$ = 0.02; omissions: *p* = 0.32, $\eta_{p}^{2}$ = 0.03).

### Performance at End of Training (Baseline)

At the end of training, all rats showed stable baseline performance at 1.2 s stimulus duration over at least three consecutive training days. All animals finished 100 trials within 30 min and reached learning criteria with accuracy >80% and omissions <20. Numbers of premature and perseverative responses were low (see Table 1).

**Table 1:** **The effect of early life maternal deprivation (MD) and mifepristone treatment on performance in the 5-choice serial reaction time task (5-choice SRTT) at the end of training (baseline)**.

	No-MD		MD	
	Vehicle	Mifepristone	Vehicle	Mifepristone
Accuracy (%)	94.08 ± 1.23	95.15 ± 0.86	93.42 ± 1.06	95.17 ± 0.77
Omissions (#)^b^*	8.00 ± 0.73	9.38 ± 0.84	7.33 ± 1.11	11.23 ± 1.41
Premature (#)	3.50 ± 1.15	5.15 ± 0.97	4.58 ± 0.58	4.50 ± 0.80
Perseverant (#)	3.75 ± 1.22	3.50 ± 0.91	5.38 ± 1.40	5.25 ± 1.18
Latency to correct (s)	0.74 ± 0.03	0.75 ± 0.03	0.74 ± 0.02	0.73 ± 0.02
Latency to reward (s)	1.12 ± 0.06	1.12 ± 0.05	1.19 ± 0.04	1.28 ± 0.07
NP pellet hole ITI (#)^a^**	302.8 ± 68.4	302.9 ± 48.4	155.0 ± 44.3	170.4 ± 30.6
NP pellet hole/TO (#)	2.33 ± 0.48	2.41 ± 0.26	1.98 ± 0.24	1.71 ± 0.24
NP stimulus holes/TO (#)^$^	0.19 ± 0.03	0.21 ± 0.03	0.32 ± 0.06	0.27 ± 0.05
Total duration (min)	18.08 ± 0.27	18.38 ± 0.23	18.33 ± 0.24	18.73 ± 0.24

Data on general performance and stimulus (stimulus holes) and goal (pellet hole) approach in no-reward periods (NP: nose pokes, ITI: inter trial interval and TO: time-out). Data represent Mean ± SEM. Group sizes: No-MD vehicle (n = 8) and No-MD mifepristone (n = 16), MD vehicle (n = 8) and MD mifepristone (n = 16). ^a^main effect of early life experience, ^b^main effect of mifepristone treatment. *p < 0.05, **p < 0.01, ^$^main effect of early life with p = 0.052.

Accuracy and premature responses at baseline level were not affected by MD (accuracy: *F*_(1,44)_ = 0.11, *p* = 0.75, $\eta_{p}^{2}$ = 0.00, premature responses: *F*_(1,44)_ = 0.05 *p* = 0.83, $\eta_{p}^{2}$ = 0.00). Mif treatment did also not affect these measures (accuracy: *p* = 0.17, $\eta_{p}^{2}$ = 0.04, premature responses: *p =* 0.44, $\eta_{p}^{2}$ = 0.01) nor did both treatments interact (accuracy: *p* = 0.74, $\eta_{p}^{2}$ = 0.00, premature responses: *p* = 0.39, $\eta_{p}^{2}$ = 0.02). Although MD did not affect omissions (*p* = 0.64, $\eta_{p}^{2}$ = 0.01), a main effect of Mif treatment on the number of omissions was observed (*F*_(1,44)_ = 4.38, *p* < 0.05, $\eta_{p}^{2}$ = 0.09), with a higher number of omissions in the Mif compared to Veh treated animals. The two treatments did not interact (*p* = 0.32, $\eta_{p}^{2}$ = 0.02). However, the number of omissions for all groups stayed well within the acquisition criteria of < 20 omissions per training session of 100 trials and performance was stable.

Differences in behavior were also seen during the no-reward periods. Compared to no-MD animals, MD animals made less nose poke responses in the pellet hole during the ITI (the interval preceding the stimulus light; *F*_(1,44)_ = 7.96, *p* = 0.01, $\eta_{p}^{2}$ = 0.15). Moreover, during time-out (the punishment period where no reward can be earned) these rats tended to approach the stimulus holes more often (*F*_(1,44)_ = 4.00, *p* = 0.052, $\eta_{p}^{2}$ = 0.08). This may suggest that MD rats show features of sign tracking behavior (Tomie et al., 1989, 2008). Mif treatment did not affect these behaviors (nose pokes in pellet hole during ITI: *p* = 0.88, $\eta_{p}^{2}$ = 0.00, nose pokes in stimulus holes during time-out *p* = 0.67, $\eta_{p}^{2}$ = 0.00), nor did it moderate the effect of MD (nose pokes in pellet hole during ITI: *p* = 0.88, $\eta_{p}^{2}$ = 0.00, nose pokes in stimulus holes during time-out: *p* = 0.42, $\eta_{p}^{2}$ = 0.02).

### Behavioral Inhibition I: Prolonged Inter-Trial Interval (7 s ITI)

**Figure 2:**
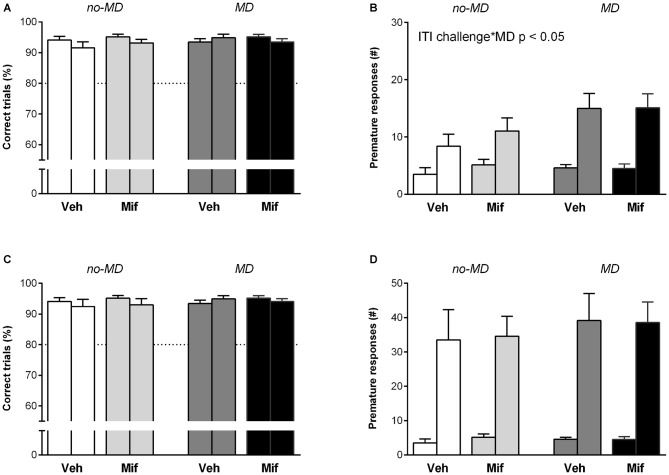
**The effect of MD and Mif on performance in the 5-choice SRTT when behavioral inhibition is challenged.** Each first bar represents baseline performance, each second bar represents test performance. Accuracy (% correct trials) and number of premature responses when **(A,B)** the inter-trial interval (ITI) is prolonged to 7 s or **(C,D)** under a randomized ITI protocol. Graphs represent Mean ± SEM. Dotted horizontal lines represent acquisition criteria. Group sizes: No-MD Veh (*n* = 8) and No-MD Mif (*n* = 16), MD Veh (*n* = 8) and MD Mif (*n* = 16). MD compared to no-MD animals made significantly more premature responses in the 7 s ITI challenge condition compared to baseline (*F*_(1,44)_ = 5.48, *p* < 0.05, $\eta_{p}^{2}$ = 0.11; **B**).

To challenge behavioral inhibition, the ITI was increased from 5 to 7 s. Animals had to wait 2 s longer before the stimulus light was shown (Figures 2A,B). The increase in ITI did not affect accuracy (*F*_(1,44)_ = 3.61, *p* = 0.064, $\eta_{p}^{2}$ = 0.08; Figure 2A). In all groups, the prolonged ITI was related to a significant increase in the number of premature responses (*F*_(1,44)_ = 52.88, *p* < 0.001, $\eta_{p}^{2}$ = 0.55; Figure 2B). A main effect of MD was seen for the number of premature responses (*F*_(1,44)_ = 5.48, *p* < 0.05, $\eta_{p}^{2}$ = 0.11); MD compared to no-MD animals made significantly more premature responses in the 7 s ITI condition. Mif treatment had no effect on the number of premature responses (*p* = 0.78, $\eta_{p}^{2}$ = 0.00), nor did it moderate the effects of MD (*p* = 0.85, $\eta_{p}^{2}$ = 0.00).

The 2 s increase in ITI led to a difference in the number of perseverative responses between Mif and Veh treated animals (*F*_(1,44)_ = 5.43, *p* < 0.05, $\eta_{p}^{2}$ = 0.11). Mif treated animals showed less perseverative responses. The number of perseverative responses was not affected by MD treatment (*p* = 0.91, $\eta_{p}^{2}$ = 0.00) and there was no interaction between MD and Mif treatment (*p* = 0.22, $\eta_{p}^{2}$ = 0.03).

Overall, the increase in ITI was a challenge for all groups, as seen by the increase in premature responses. The higher increase in premature responses in the MD groups suggests less behavioral inhibition in animals with a background of early life stress.

### Behavioral Inhibition II: Random Inter-Trial Interval (5, 7, 10, 13 and 15 s ITI)

The second test to challenge behavioral inhibition consisted of trials with 5, 7, 10, 13 or 15 s ITIs that were presented in a pseudo-random order over 100 trials. Presenting animals with a random ITI was a considerable challenge, as can be seen from the significant overall increase in premature responses (*F*_(1,44)_ = 90.45, *p* < 0.001, $\eta_{p}^{2}$ = 0.67; Figures 2C,D). However, the increase in premature responding was not affected by MD (*p* = 0.50, $\eta_{p}^{2}$ = 0.01), Mif (*p* = 0.94, $\eta_{p}^{2}$ = 0.00), nor their interaction (*p* = 0.99, $\eta_{p}^{2}$ = 0.00). Accuracy was not affected by random ITI (*F*_(1,44)_ = 0.91, *p* = 0.35, $\eta_{p}^{2}$ = 0.02).

In response to a random ITI, the latency to correct responses increased (*F*_(1,44)_ = 15.63, *p* < 0.001, $\eta_{p}^{2}$ = 0.26), while the latency to collect rewards from the pellet hole decreased (*F*_(1,44)_ = 10.71, *p* < 0.01, $\eta_{p}^{2}$ = 0.20) in all groups. These measures were not influenced by MD (latency to correct: *p* = 0.88, $\eta_{p}^{2}$ = 0.00; latency to reward: *p* = 0.23, $\eta_{p}^{2}$ = 0.03) or Mif treatment (latency to correct: *p* = 0.72, $\eta_{p}^{2}$ = 0.00; latency to reward: *p* = 0.09, $\eta_{p}^{2}$ = 0.06), neither did the two treatments interact (latency to correct: *p* = 0.32, $\eta_{p}^{2}$ = 0.02; latency to reward: *p* = 0.57, $\eta_{p}^{2}$ = 0.01).

As observed with the 7 s ITI, a main effect of Mif treatment was observed in the number of perseverative responses (*F*_(1,44)_ = 5.55, *p* < 0.05, $\eta_{p}^{2}$ = 0.11). Mif treated animals showed a decrease in perseverative responses, while the perseverative responses in Veh treated animals did not change compared to baseline. The number of perseverative responses was not affected by MD (*p* = 0.84, $\eta_{p}^{2}$ = 0.0), nor did MD moderate the effect of Mif (*p* = 0.28, $\eta_{p}^{2}$ = 0.03).

### Attention I: Short Stimulus Duration (0.5 s)

**Figure 3:**
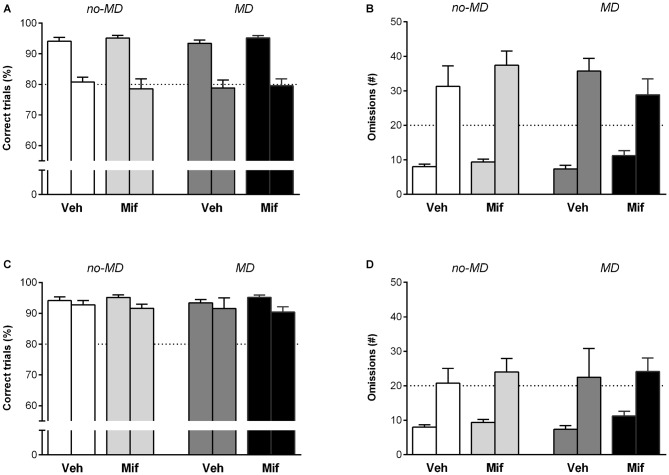
**The effect of MD and Mif on performance in the 5-choice SRTT when attentional load is increased.** Each first bar represents baseline performance, each second bar represents test performance. Accuracy (% correct trials) and number of omissions when **(A,B)** the stimulus duration is shortened to 0.5 s or **(C,D)** when a novel object is introduced in the cage. Graphs represent Mean ± SEM. Dotted horizontal lines represent acquisition criteria. Group sizes: No-MD Veh (*n* = 8) and No-MD Mif (*n* = 16), MD Veh (*n* = 8) and MD Mif (*n* = 16).

Attention was challenged with a stimulus duration of 0.5 s as compared to 1.2 s during training. Overall, a shorter stimulus duration led to a decrease in accuracy (*F*_(1,44)_ = 97.33, *p* < 0.001, $\eta_{p}^{2}$ = 0.69) and an increase in omissions (*F*_(1,44)_ = 99.70, *p* < 0.001, $\eta_{p}^{2}$ = 0.69; Figures 3A,B). An increase in premature responses (*F*_(1,44)_ = 10.61, *p* < 0.01, $\eta_{p}^{2}$ = 0.19) was also observed (data not shown). In addition, animals became quicker to make correct responses and collect rewards from the pellet hole (latency to correct *F*_(1,44)_ = 123.63, *p* < 0.001, $\eta_{p}^{2}$ = 0.74, and latency to rewards *F*_(1,44)_ = 28.54, *p* < 0.001, $\eta_{p}^{2}$ = 0.39,). None of these measures were influenced by MD or Mif treatment, nor did these treatments interact (accuracy: *p* = 0.96; 0.49; 0.72, $\eta_{p}^{2}$ = 0.00; 0.01; 0.00, omissions: *p* = 0.59; 0.54; 0.12, $\eta_{p}^{2}$ = 0.01; 0.01; 0.06, premature responses: *p* = 0.14; 0.83; 0.81, $\eta_{p}^{2}$ = 0.05; 0.00; 0.00, latency to correct: *p* = 0.31; 0.54; 0.63, $\eta_{p}^{2}$ = 0.02; 0.01; 0.01, latency to rewards: *p* = 0.21; 0.46; 0.28, $\eta_{p}^{2}$ = 0.04; 0.01; 0.03 for MD, MIF and interaction effects respectively; Figures 3A,B).

### Attention II: Introducing a Novel Object (Woodblock)

During the second attention challenge, a novel object (woodblock) was introduced in the operant chamber. This led to an overall decrease in accuracy (*F*_(1,44)_ = 6.97, *p* = 0.01, $\eta_{p}^{2}$ = 0.14) and increase in omissions (*F*_(1,44)_ = 29.14, *p* < 0.001, $\eta_{p}^{2}$ = 0.40; Figures 3C,D). The latency to make correct responses and the latency to collect rewards from the pellet hole increased (latency to correct *F*_(1,44)_ = 31.89, *p* < 0.001, $\eta_{p}^{2}$ = 0.42 and latency to rewards *F*_(1,44)_ = 102.17, *p* < 0.001, $\eta_{p}^{2}$ = 0.70). Again, none of these measures were influenced by MD or MIF treatment, nor did these two treatments interact (accuracy: *p* = 0.69; 0.26; 0.88, $\eta_{p}^{2}$ = 0.00; 0.03; 0.00; omissions: *p* = 0.95; 0.97; 0.88, $\eta_{p}^{2}$ = 0.00; 0.00; 0.00; latency to correct: *p* = 0.69; 0.94; 0.76, $\eta_{p}^{2}$ = 0.00; 0.00; 0.00; latency to rewards: *p* = 0.26; 0.80; 0.99, $\eta_{p}^{2}$ = 0.03; 0.00; 0.00 for MD, MIF and interaction effects respectively; Figures 3C,D).

## Discussion

The present study aimed to specifically investigate the long-term effects of early life stress and early adolescent mifepristone treatment on behavioral inhibition and attention in the 5-choice SRTT. We demonstrated that 24 h MD on PND 3 significantly reduces behavioral inhibition, as evident from a modest increase in premature responses when the inter-trial-interval was prolonged. MD did not influence attention in this task, since no differences in behavior were seen between the maternally deprived and non-deprived groups when the stimulus duration was shortened or with the introduction of a novel object into the operant chamber. A 3-day Mif treatment during early adolescence did not mitigate the effects of MD on behavioral control, but by itself slightly reduced perseverative behavior.

### Effects of 24 h Maternal Deprivation

Five days after weaning, MD rats weighed less than no-MD controls, which was also found by others after comparable severe early life conditions (Burke et al., 2013; Fuentes et al., 2014; Marco et al., 2015). This emphasizes that the lack of maternal care during 24 h represents both a lack of physical care (contact, licking and grooming) and a lack of nutrition. It is not unlikely that the observed effects of MD are at least in part caused by a combination of the two factors and with the current protocol these two factors cannot be addressed separately. The attenuation in weight gain lasted until the start of 5-choice SRTT training when animals were 12 weeks of age. The effect of MD on body weight was not affected by Mif treatment on PND 26–28. Because from week 12 onwards animals were food restricted and maintained on 90–95% of their body weight and their body weights at the start of training was no longer a reflection of their natural free-fed weight. We cannot entirely exclude that body weight differences might have affected acquisition of the 5-choice task, but this seems unlikely since all animals acquired the task equally well.

When tested for behavioral inhibition by increasing the ITI duration from 5 to 7 s, MD animals proved to be more impulsive. That is, MD compared to no-MD rats had a significantly higher increase in premature responding. The current findings are in accordance with those of Lovic et al. (2011a), where artificially reared rats showed increased impulsive action but not impulsive choice in adulthood. Fuentes et al. (2014) tested rats subjected to a combination of early life maternal separation and restriction of bedding material in a 5-choice SRTT protocol that is comparable with our training and baseline performance. In terms of 5-choice SRTT acquisition our results are similar to their findings, i.e., we also report no effects of early life stress on impulsive responding during acquisition and baseline performance. However, Fuentes et al. (2014) did not challenge behavioral inhibition by manipulation of the ITI. It was exactly in this condition that in our experiments MD effects became apparent. Such effects of early life stress might thus have remained unnoticed in the Fuentes et al. (2014) study. It should be noted that random variation in ITI from 5 to 15 s elicited very high levels of premature responses in all animals. This “ceiling” effect may have masked group differences in our second test of behavioral inhibition.

Important neural substrates involved in impulsivity as measured by the 5-choice SRTT include the nucleus accumbens, prefrontal cortex, hippocampus, amygdala, ventral tegmental area and locus coeruleus (Dalley et al., 2008, 2011). In the prenatal and early postnatal period, the prefrontal cortex, hippocampus and amygdala are highly plastic and susceptible to the programming effects of glucocorticoids (Lupien et al., 2009), which could explain the effects of MD in this task. Next to increased premature responding when the ITI is prolonged, MD animals made fewer responses in the pellet hole in periods when no reward could be obtained (ITI and time-out), and were more focused on the stimulus holes. These behaviors are features of sign tracking animals: sign tracking occurs when incentive salience is attributed to a conditioned stimulus that is associated with a reward, be it natural or drugs (Tomie et al., 1989, 2008). The neural substrates that regulate sign tracking show considerable overlap with the brain regions that are involved in impulsive behavior (Tomie et al., 2008). Indeed, animals that were classified as sign-trackers on a Pavlovian conditioned approach task were also more impulsive in a 2-choice SRTT that measured impulsive action (Lovic et al., 2011b). Moreover, social isolation rearing from PND 5 onwards resulted in sign tracking during Pavlovian conditioned approach testing (Lomanowska et al., 2011).

Recently, in a parallel study, we showed that a complex rearing environment from adolescence onwards impairs behavioral inhibition but improves attention of rats in the 5-choice SRTT (van der Veen et al., 2015). Rats that were reared from PND 26 onwards in groups of 10 in enriched Marlau^TM^ cages were tested on the 5-choice SRTT and compared to pair-housed rats in standard type III Makrolon cages. Furthermore, the influence of early life experiences on the effects of complex housing were tested, by comparing PND 3 MD rats with no-MD rats, either complex or standard housed. In contrast to our current findings, there was no evidence for an effect of MD on either attention or behavioral inhibition. An important difference with the current study is that van der Veen et al. (2015) used standard housing as a control condition for complex housing, either with or without MD on PND 3, while in the current study the control condition for Mif treatment consisted of Veh treatment on PND 26–28, with or without MD op PND 3. Thus, the administration of Veh (or Mif) through oral gavage twice daily for 3 days in early adolescence may have amplified the effects of earlier experienced MD. Administration of drugs through oral gavage is a quick and painless intervention that is not considered highly stressful. However, during the treatment period the home cage situation is disturbed twice daily for 3 consecutive days, and animals have to be restrained, if only briefly. It is thus not unlikely that the combined procedure was experienced as stressful by the animals. Taking the results of the two studies together, the oral gavage treatment may be considered a second “hit” during pre-puberty that is necessary for the behavioral impairments caused by MD to become apparent. This would be in line with other studies showing effects of cumulative stress on adult phenotype (McEwen, 1998; Daskalakis et al., 2013). A fact that may have contributed to the differences in MD findings between the two studies is the large effect of complex housing in the van der Veen et al. (2015) study, which could have masked the relatively subtle MD effects.

When attentional load was increased by either a shorter stimulus duration or the introduction of a novel object into the operant chamber, animals responded, as expected, with a decrease in accuracy and an increase in omissions. The shorter stimulus time was the most challenging attention task, since decreases in accuracy and increases in omission were strongest for this protocol. However, attention was not differentially affected by MD. Therefore, although impaired attention is a core feature in some of the psychiatric diseases linked to early life adversity we saw no attentional impairments in the current study.

### Effects of Early Adolescent Mifepristone Treatment

It is thought that the detrimental effects of early life stress originate from glucocorticoid overexposure in brain regions that at the time of stress are still in development and therefore sensitive to the programming effects of glucocorticoids. As these brain regions, particularly the prefrontal cortex (Fuster, 2001), still undergo important developmental changes in adolescence, we aimed to counteract the effects of glucocorticoid overexposure by treating the animals with the GR antagonist Mif (Johanssen and Allolio, 2007) during early adolescence. We hypothesized that selectively blocking the GR, in favor of MR activation, for a 3-day period early in adolescence would be a potential method to restore the MR:GR imbalance and thereby behavioral deficits related to early life stress. Previously this intervention was found to reverse the MD-induced reduction of hippocampal neurogenesis (Loi et al., 2014; and unpublished observations). In the current study, however, Mif treatment did not counteract the impairment in behavioral inhibition observed after MD. We cannot exclude that the selected window and duration of intervention are effective for properties related to the hippocampus, but not for preventing or normalizing effects of MD on impulsivity.

Interestingly, while Mif was ineffective in moderating the effects of MD, we did find an effect of Mif treatment by itself on perseverative responses; i.e., Mif treated animals made less perseverative responses than Veh treated animals. A high number of perseverative responses in this task is thought to reflect compulsive behavior (Dalley et al., 2011) and this would suggest that Mif might act to prevent this type of behavior. However, the relevance of this finding should be interpreted with caution since perseverative responses were quite low in all groups. The lower perseverance in animals where MR:GR activation was increased through GR blockade would however fit with the behavioral perseverance observed in radial maze performance of mice with a loss of forebrain MRs (Berger et al., 2006). Protocols specifically addressing this type of behavior would be required to shed more light on the present results.

Overall, the results of this study suggest that a separation from the mother for 24 h on PND 3, during the stress hyporesponsive period, impacts on impulsivity, but does not influence attention as measured in the 5-choice SRTT; blockade of GRs for 3 days during early adolescence was ineffective to counteract the behavioral changes caused by early life stress. Interestingly, comparison with earlier results suggests that mildly stressful experiences during adolescence may be necessary to reveal effects of early life stress. If so, this would support the notion that early life adverse conditions do not necessarily cause behavioral deficits in adulthood, but that multiple “hits” may compromise the adaptive capacity of individuals (McEwen, 1998; Daskalakis et al., 2013).

## Author Contributions

Authors have made substantial contributions to the following: conception and design of the study: RvdV, MHvIJ, MJB-K, MJ. Interpretation of data: RvdV, JK, MHvIJ, MJB-K, MJ. Acquisition of data: RvdV, JK, LvdT, ML. Analysis of data: JK, RvdV, MHvIJ, MJB-K, MJ. Drafting the article critically for important intellectual content: JK, RvdV, MHvIJ, MJB-K, MJ. Final approval of the version to be submitted: JK, RvdV, LvdT, ML, MHvIJ, MJB-K, MJ. Agreement to be accountable for all aspects of the work in ensuring that questions related to the accuracy or integrity of any part of the work are appropriately investigated and resolved: JK, RvdV, LvdT, ML, MHvIJ, MJB-K, MJ.

## Conflict of Interest Statement

The authors declare that the research was conducted in the absence of any commercial or financial relationships that could be construed as a potential conflict of interest.
